# Vibration Fatigue Damage Estimation by New Stress Correction Based on Kurtosis Control of Random Excitation Loadings

**DOI:** 10.3390/s21134518

**Published:** 2021-07-01

**Authors:** Yuzhu Wang, Roger Serra

**Affiliations:** 1 INSA Centre Val de Loire, Laboratoire de Mécanique G. Lamé E.A.7494, Campus de Blois, Equipe DivS, 3 rue de la Chocalaterie, 41000 Blois, France; yuzhu.wang@insa-cvl.fr; 2 China Academy of Railway Sciences, Locomotive Car Research Institute, Beijing 100081, China

**Keywords:** vibration fatigue test, non-gaussian excitation, kurtosis control

## Abstract

In the pioneer CAE stage, life assessment is the essential part to make the product meet the life requirement. Commonly, the lives of flexible structures are determined by vibration fatigue which accrues at or close to their natural frequencies. However, existing PSD vibration fatigue damage estimation methods have two prerequisites for use: the behavior of the mechanical system must be linear and the probability density function of the response stresses must follow a Gaussian distribution. Under operating conditions, non-Gaussian signals are often recorded as excitation (usually observed through kurtosis), which will result in non-Gaussian response stresses. A new correction is needed to make the PSD approach available for the non-Gaussian vibration to deal with the inevitable extreme value of high kurtosis. This work aims to solve the vibration fatigue estimation under the non-Gaussian vibration; the key is the probability density function of response stress. This work researches the importance of non-Gaussianity numerically and experimentally. The beam specimens with two notches were used in this research. All excitation stays in the frequency range that only affects the second natural frequency, although their kurtosis is different. The results show that the probability density function of response stress under different kurtoses can be obtained by kurtosis correction based on the PSD approach of the frequency domain.

## 1. Introduction

The random vibration load closed to the mechanical components’ natural frequencies will significantly affect their fatigue life. It is called vibration fatigue, which has become a hot subject in recent years. For the fatigue-life estimation of random vibration, two methods are usually used: time domain and frequency domain [1].

For the time-domain approach, the response-stress distribution and its number of cycles are estimated through cycle counting. The cumulative fatigue damage is determined by Miner’s law with the Wohler’s curve. It can be understood that at one stress level, the number of cycles used is the damage. The total number of cycles that can be used is the fatigue life. The computational power it needs is proportional to the amount of data. The frequency-domain approach described random vibration by power spectral density (PSD), which describes the power distribution along with the frequency content of the process. The second-order spectrum analysis in the frequency domain can better study the mechanical system excited by stochastic stationary Gaussian vibration. In the aspect of signal extrapolation, the frequency-domain approach has advantages, because the frequency distribution is not affected by signal duration. However, the dynamic behavior of the mechanical system is linear in theory, so the stress process also has a Gaussian distribution. In this manner, the response–stress distribution can be performed using the stress PSD, which is the output of the dynamic analysis, through one of the frequency methods present in the literature [2,3,4,5,6]. As well-known, the evaluation of an expected value of any random parameter derived from PSD is equivalent to estimating the same parameter in the unlimited time history in the random-stress process. Thus, it can be considered that a fatigue-damage estimation performed in the frequency domain significantly reduces the computational times. For this reason, an extension of the preliminary hypotheses of the frequency-domain approach may be well received. 

In a real case, however, the typical loading is non-Gaussian, and this can cause the response to be non-Gaussian, which may lead to shorter fatigue life. This means that sometimes the frequency-domain approach for fatigue estimation directly performed by stress PSD cannot be applied. For this reason, non-Gaussian vibration fatigue has been the focus. Typical non-Gaussian loads include the excitation of rail irregularity and pressure fluctuations for an aircraft. It is proposed in military environmental standards that the non-Gaussian loads case under extreme conditions need to be considered [7,8]. There are several studies that were published in recent years to show how non-Gaussian excitation impacts vibration fatigue life. In literature, Kihl et al. first proposed that the fatigue damage caused by the stress-time history can be determined by the Gaussian damage *D_g_,* which is obtained from the methods mentioned above and the corrective coefficient of non-Gaussianity *λ_ng_* [9]:

(1) $$D_{ng}=\lambda_{ng}D_{g}$$

Winterstein obtained a polynomial to generate non-Gaussian time histories. Then he obtained *λ_ng_* through the Wohler’s curve of the component and parameters from the polynomial [10]. 

Benasciutti made it so that the non-Gaussian loading was modeled as a transformed Gaussian process and defined *λ_ng_* with kurtosis, skewness and material parameter, also the parameters which are used to generate non-Gaussian excitation [11,12]; Rizzi et al. and Kihm et al. describe how non-Gaussian excitation affects the fatigue life of linear and nonlinear structures [13,14,15]. Braccesi et al. propose a corrective coefficient to the narrow-band formula in their research. He points the most significant field of the research consists in the evaluation of non-normality indices without stress-time history reconstruction [16]. Nieslony verifies the case of a non-zero mean stress, non-Gaussian loading histories with the use of the spectral method with the corrective factor and obtains successful results. The only difference in *λ_ng_* is the power of the material parameter [17]. Palmieri et al. point out that fatigue life due to burst and non-stationary excitations is significantly shorter [18].

It is a real challenge to obtain the kurtosis of the response stress at the critical position only through the frequency-domain information without constructing the time-domain signal, which makes the use of this method problematic. It is necessary to try to achieve this goal through the kurtosis of the excitation signal rather than the kurtosis of response stress. Although it is pointed out in the above literature that *λ_ng_* is only related to the property of the material and the kurtosis and the skewness of excitation, no other material is used to verify it. The author considers that a single corrective coefficient cannot reflect the influence of non-Gaussian methods on fatigue-damage estimation. At high kurtosis, the extreme value will aggravate the fatigue damage process and further shorten the fatigue life. According to previous studies, kurtosis also has an effect on other links of fatigue-damage estimation. For a more accurate response to fatigue damage, the breakthrough point of the study was chosen to be the cycle counting of response stress.

In the present study, a series of finite duration data with the same PSD is designed to demonstrate the effect of non-Gaussianity (kurtosis) on the response-stress distribution. The PSD shape and frequency range never change in this study. The assumption is maintained that the dynamic behavior of the mechanical system is linear and the excitation is stationary. The aim of this study is to experimentally research the improved PSD method in the frequency domain to infer the distribution of response stress under excitation with different kurtoses. The advantage of this method is that it can directly reflect the range of stress changes. Ultimately, more accurate damage can be estimated.

This manuscript is organized as follows: in Section 2, the PSD approach in the frequency for the response of the dynamic structure under vibration is presented. In Section 3 and Section 4, the corrected PSD method is proposed for non-Gaussian vibration based on kurtosis control and demonstrated. In Section 5, this method is verified under different PSD level signals. In this paper, stress-distribution correction rather than damage correction is used to solve the fatigue-damage estimation for non-Gaussian random vibration. It was verified that the method proposed in this study can solve the fatigue-damage estimation at different kurtosis levels with higher accuracy than previous methods.

## 2. PSD Approach in the Frequency Domain

Now, when calculating the general method of fatigue damage, according to Wohler’s curve of the material, calculate the damage under the corresponding stress cycle and add it to the total damage suffered by the structure. This result is based on the established Miner’s law [19]. Therefore, obtaining the damage under the corresponding stress cycle is called the key to calculate the fatigue damage. The more commonly used methods are the rainflow counting method based on the time-domain signal (time-domain method) and the PSD method based on the frequency-domain information (frequency-domain method).

The power spectral density (PSD) is a description of the power contained in the frequency components of the random vibration signal and is a statistical characteristic in the frequency domain of random vibration [2,20]. In random vibration, due to the time history of vibration being significantly non-periodic, the power spectral density must be used to calculate. The random vibration signal can be regarded as composed of an infinite number of simple harmonic motions, so the power spectrum of the random vibration signal is the sum of the harmonic vibration power in a given frequency range. For a time-series *x(t)* of a classic random signal with zero mean, using PSD *S(ω)* to represent its frequency-domain characteristic, $R_{x}\left(\tau \right)$ is the autocorrelation function of x(t):

(2) $$S_{x}\left(\omega \right)=\frac{1}{2\pi }\int_{-\infty }^{\infty }R_{x}\left(\tau \right)e^{-i\omega \tau }d\tau \;$$

The power spectral density at the specified frequency is the average of the mean square values of the signal.

For linear structures, the vibration of a multi-degree of a freedom system can be described as [21]:

(3) $$M\ddot{x}+C\dot{x}+Kx=f\;$$

where *M* is the mass matrix, C is the damping matrix, *K* is the stiffness matrix, x is the vector of degrees of freedom and f is the excitation vector. By constructing the modal matrix of the system and using the modal superposition method, the displacement mode shape can be obtained. Then, the frequency-response equation of structural stress is obtained by the relationship between displacement and stress [22,23,24]:

(4) $$H^{\sigma }\left(\omega \right)=\overset{n}{\underset{i=1}{\sum }}\frac{\varphi_{i}^{\sigma }\varphi_{i}^{T}}{k_{i}+\omega^{2}m_{i}+j\omega c_{i}}$$

where *H^σ^*(*ω*) denotes the stress-frequency-response function and *k_i_, m_i_, c_i_* are the *i*-th modal stiffness, modal mass and modal damping, respectively. $\varphi_{i}^{\sigma }$ is the corresponding modal strain component of the system.

In this project, the stress PSD function $S_{b}\left(f\right)$ at the structural hazard point is determined by the following function:

(5) $$S_{b}\left(f\right)=A_{a}\left(f\right)H_{ba}^{2}\left(f\right)$$

$A_{a}\left(f\right)$ is excitation applied to point a and $H_{ba}^{}\left(f\right)$ is the frequency-response function at point *b*, and the structure excitation is at point *a*. The N-order moment of inertia is defined as

(6) $$m_{n}=\overset{\infty }{\underset{i=1}{\sum }}f_{i}^{n}S_{b}\left(f_{i}\right)\Delta f$$

The root mean square (RMS) values of the stress response $\sigma_{RMS}$ and the input excitation obtained by the 0-order moment of inertia are

(7) $$\sigma_{RMS}=\sqrt{m_{0}}=\sqrt{\overset{+\infty }{\underset{i=1}{\sum }}A_{a}(f_{i})H_{ba}^{2}\left(f\right)\Delta f}$$

The frequency domain usually uses the moment of inertia, the approximate estimate, the zero-value positive pass expectation *E*(0) and the peak expectation *E*(*p*):

(8) $$E\left(0\right)=\sqrt{m_{2}/m_{0}}$$

(9) $$E\left(p\right)=\sqrt{m_{4}/m_{2}}$$

In the frequency domain, the random process is usually described using the spectral irregularity factor α and the spectral width factor ε:

(10) α=E(0)/E(p)

(11) $$\varepsilon =\sqrt{1-\alpha^{2}},\;\;\;\varepsilon \in \left(0,1\right)$$

Dirlik used Monte Carlo to simulate the power-spectral density function in 70 different shapes and proposed an exponential distribution and two Rayleigh distributions to describe the probability density function of approximate rain flow cyclic amplitude. The Dirlik method has high accuracy [4,25,26].

The rainflow cycle amplitude probability density estimate is given by:

(12) $$p{\left(S\right)}^{DK}=\frac{1}{2\sqrt{m_{0}}}\left[\frac{G_{1}}{Q}exp\left(-\frac{Z}{Q}\right)+\frac{G_{2}Z}{R^{2}}exp\left(-\frac{Z^{2}}{2R^{2}}\right)+G_{3}Zexp\left(-\frac{Z^{2}}{2}\right)\right]$$

where *Z* is the normalized amplitude and *Xm* is the mean frequency, as fellow

$$Z=\frac{S}{2\sqrt{m_{0}}}\;\mathrm{and}\;x_{m}=\frac{m_{1}}{m_{0}}{\left(\frac{m_{2}}{m_{4}}\right)}^{\frac{1}{2}}$$

and other parameters are defined as:

$$G_{1}=\frac{2\left(x_{m}-\alpha_{2}^{2}\right)}{1+\alpha_{2}^{2}},\;G_{2}=\frac{1-\alpha_{2}-G_{1}+G_{1}^{2}}{1+R},\;G_{3}=1-G_{1}-G_{2}$$

$$R=\frac{\alpha_{2}-x_{m}-G_{1}^{2}}{1-\alpha_{2}-G_{1}+G_{1}^{2}}\;\mathrm{and}\;Q=\frac{1.25\left(\alpha_{2}-G_{3}-G_{2}R\right)}{G_{1}}$$

For Gaussian signals, the stress-probability density equation obtained from the PSD and rainflow count results obtained under the time-domain signal should be consistent. From Wohler’s curve of the material, structural damage can be determined. The classical method is the linear damage accumulation principle proposed by Miner’s Law, which is:

(13) $$n\left(S\right)=E\left(0\right)p\left(S_{i}\right)\Delta S_{i}$$

The whole life under the corresponding stress level can be obtained from Wohler’s curve of the material. Through Miner’s law, the damage corresponding to the stress level can be obtained.

(14) $$D=\sum^{\;}\frac{n\left(s\right)}{N\left(s\right)}$$

## 3. Non-Gaussian PSD Method in the Frequency Domain

Generally, a random vibration signal *x* is distinguished as a Gaussian signal if its probability density function *P(x)* is given as

(15) $$P\left(x\right)=\frac{1}{\sqrt{2\pi \sigma^{2}}}e^{\frac{{\left(x-\mu \right)}^{2}}{2\sigma^{2}}}$$

where *μ* is the mean and σ is the standard deviation. The *i*-th central moment was defined as

(16) $$M_{i}=\frac{1}{n}\overset{n}{\underset{j=1}{\sum }}{\left(x_{j}-\mu \right)}^{i}$$

*n* is the number of data points related to time and sample rate. Commonly, the second-order central moment (*i* = 2) *M_2_* is the same as variance *σ*^2^.

Two parameters are used to determine whether random variables follow Gaussian distribution, skewness *S* and kurtosis *K*, which are defined as follows:

(17) $$S=\frac{M_{3}}{\sigma^{4}}\;\mathrm{and}\;K=\frac{M_{4}}{\sigma^{4}}$$

The skewness is an index of asymmetry of the mean value. The kurtosis is an index of sharpness. Generally, the contribution of skewness in fatigue-damage estimation is tiny, and it is a common practice to focus only on kurtosis.

As discussed in the first section of this paper, Braccesi’s method is more acceptable than other methods. In this paper, this method is selected for comparison [16]. *m* is the coefficient of Wohler’s curve:

(18) $$\lambda_{ng}=exp\left(\frac{m^{\frac{3}{2}}}{\pi }\left(\frac{K-3}{5}-\frac{S^{2}}{4}\right)\right)$$

According to the 1986 Moors’ paper, kurtosis is actually used to represent the dispersion of the data between the mean ± RMS [27]. High kurtosis values will appear for two reasons:

1. Most data concentrated around the mean, but also contain occasional values far from the mean;
2. The trailing distribution weight is too large.

On the contrary, higher kurtosis means that there is a sizeable extreme value. For the same PSD response stress, the higher the kurtosis, the higher the extremum. The fatigue characteristics of materials, Wohler’s curve, has a high sensitivity to stress, especially on the left side of the curve; with the increase of stress, life decreases sharply. Therefore, the author’s view is that a single corrective coefficient cannot accurately reflect the influence of high kurtosis (higher than 5) on the fatigue-damage estimation under non-Gaussian random vibration. Moreover, the maximum stress may exceed the yield limit or even fracture limit at high kurtosis. Besides, the nonlinear damage-accumulation principle needs to be considered due to the influence of the loading sequence at high kurtosis [28]. Therefore, it is necessary to obtain the probability distribution function of response stress under non-Gaussian random vibration.

So, the stress distribution function in the non-Gaussian frequency-domain method can be hypothesized as

(19) $$p{\left(s\right)}_{NG}=\alpha p{\left(\beta \left(s-\Delta S_{c}\right)\right)}_{G},\;s\in \left(0,pRMS\right)$$

The commonly used stress range 0–6 RMS becomes 0–*p* RMS. Among them, *α* is the maximum distribution adjustment value, also called enlarge scale; *β* is the *α* concentration adjustment coefficient; $\Delta S_{c}$ is the center offset and *p* is the stress maximum-range coefficient. Through parameter-fitting, the relationship between these four parameters and kurtosis is established.

## 4. Virtual Experiment

**Figure 1:**
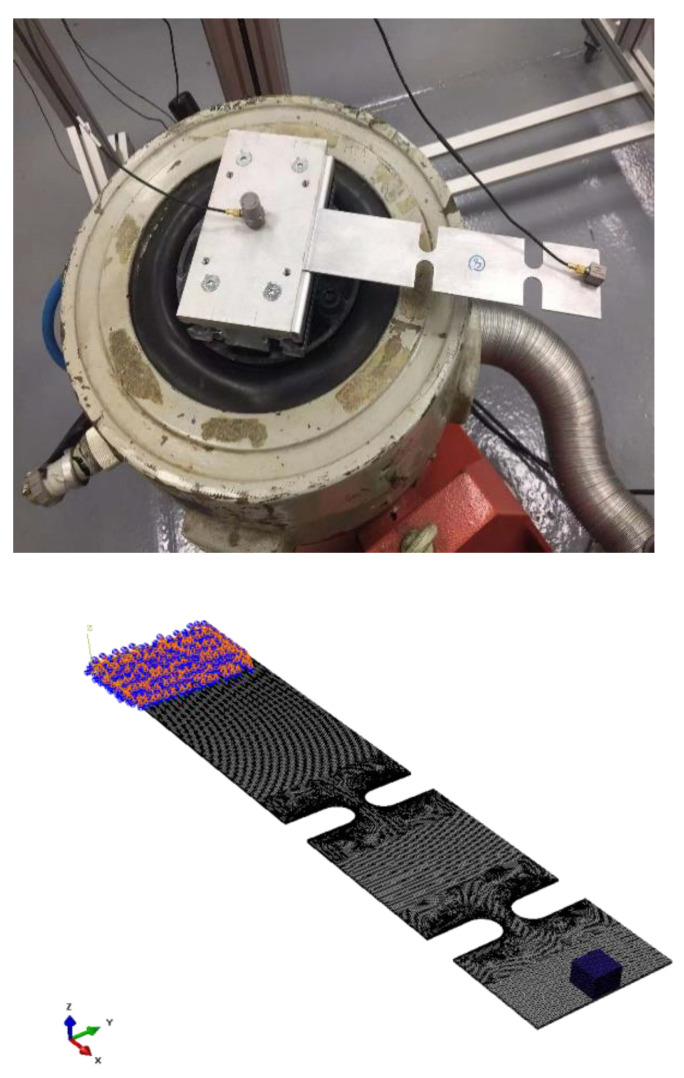
The experiment system and the virtual experiment of two notch specimens.

In order to highlight the differences from the kurtosis effect and avoid the interference of other factors, the virtual test system is used to repeat the response process of specimens under the excitation of non-Gaussian signals. The virtual test system uses actual test data to build a finite element specimen and adjusts the material density, stiffness and damping coefficients to make the entire model match the real situation. Double-notched specimens are used as research objects in this study. The experiment system is shown in Figure 1. The material card of the specimen shown in Table 1. Through the modal analysis for the specimen both experiment and virtual experiment, the density and modal information of the specimens are obtained.

According to the modal analysis result, the second-order mode 88 Hz is selected and the frequency radius of the excitation is 40 Hz. In the beginning, 30 min total length time series of acceleration were generated. They have the same PSD level and form but different kurtosis. Here, a relatively safe PSD level is selected to facilitate the observation of damage at high kurtosis. The sample rate is 512 Hz according to the Shannon formula. Due to the computational power, the excitation signal with a length of 10 min is intercepted from each datum and it is the minimum that can pass the ergodicity test. Eight different kurtosis levels are studied, from three to twenty and two cases for each kurtosis level. The tolerance of the kurtosis is ±0.4. At this stage, a total of 16 time series are created. They have the same frequency domain information, as shown in Table 2.

**Figure 2:**
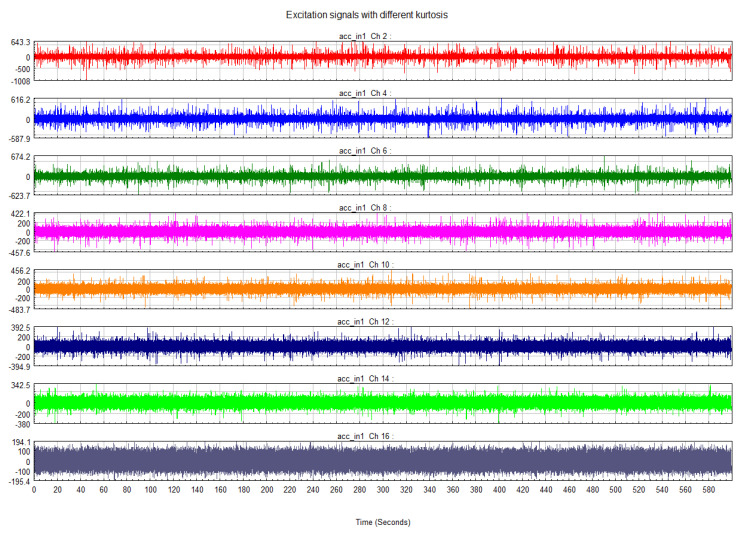
Excitation signal (10 min) with kurtosis (20, 10, 8, 5, 4.5, 4, 3.5, 3).

**Figure 3:**
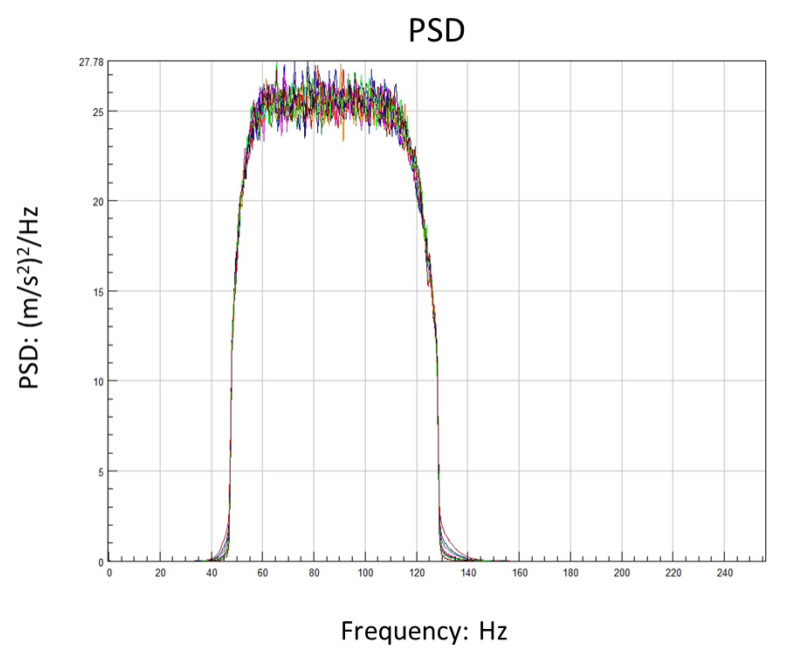
PSD of excitation acceleration in each kurtosis.

From Figure 2, the difference in the maximum values of the signals with different kurtosis is obvious. Figure 3 shows that the energy distribution of the signal is consistent in the frequency domain. The details of each signal are shown in Table 3.

In the process of the virtual experiment, through finite element simulation by Abaqus, all parameters are verified in the reality modal analysis process with the specimen data. Wohler’s curve of the specimen is also checked in order to reduce the possible difference from manufacturing [29].

**Figure 4:**
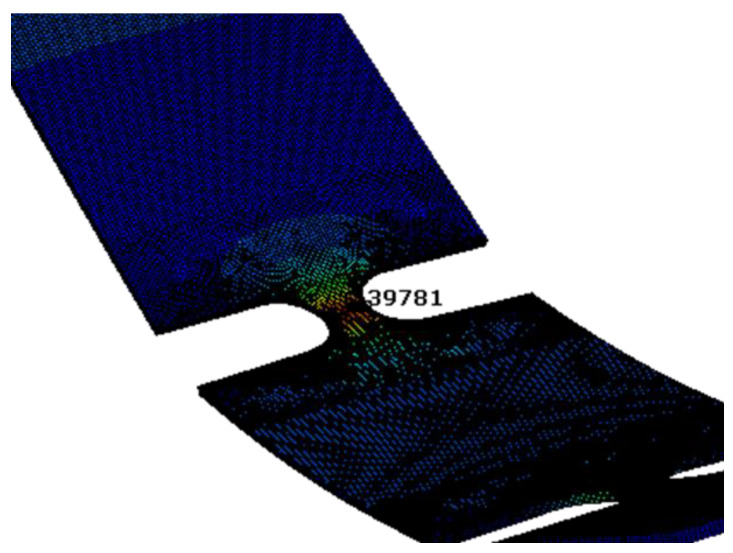
Critical node of the FE model.

The maximum response stress occurred at the center section of the first notch. One of the four nodes is selected as the observation object, shown in Figure 4. Although it is mentioned in the article by George and Michalis that materials will exhibit a non-linear response under non-Gaussian excitation, temporarily this effect is ignored in this study [30].

**Figure 5:**
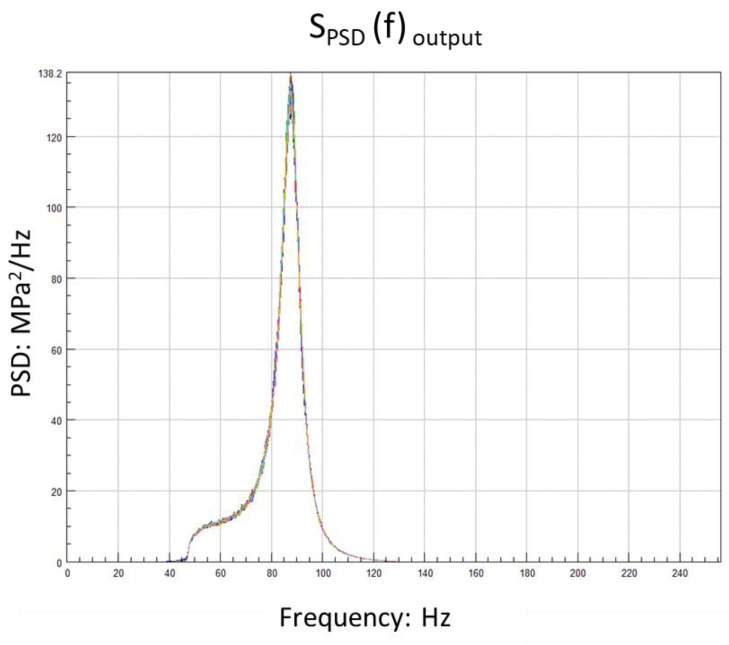
PSD of response stress in each kurtosis.

From the response-stress results shown in Table 4, it was found that the response stress’s kurtosis was attenuated. As the acceleration of kurtosis increases, the kurtosis of the response stress decays more significantly. The RMS is 42.86 MPa. There is no significant effect from kurtosis on the RMS and PSD. The details are shown in Figure 5.

By comparing the stress-cycle distribution results of different kurtoses, it can be found that as kurtosis increases, the peak of the distribution function increases; the highest point shifts to the low-stress region, and the maximum stress distribution range increases [31]. However, according to research, the maximum and minimum values of response stress are related to the value of the input signal, which means that it is determined by the value of the excitation acceleration. It can be concluded that the magnitude of the response stress is related to the input kurtosis. Therefore, in the relationship between kurtosis and damage in subsequent studies, kurtosis refers to the input kurtosis, that is, the kurtosis of the excitation acceleration. In addition, most of the time, it is more difficult to obtain the response stress than excitation.

Through the rainflow counting method, the probability density distribution of response stress can be obtained. Here we also need to pay attention to the maximum stress. It accounts for a tiny proportion but cannot be ignored.

**Figure 6:**
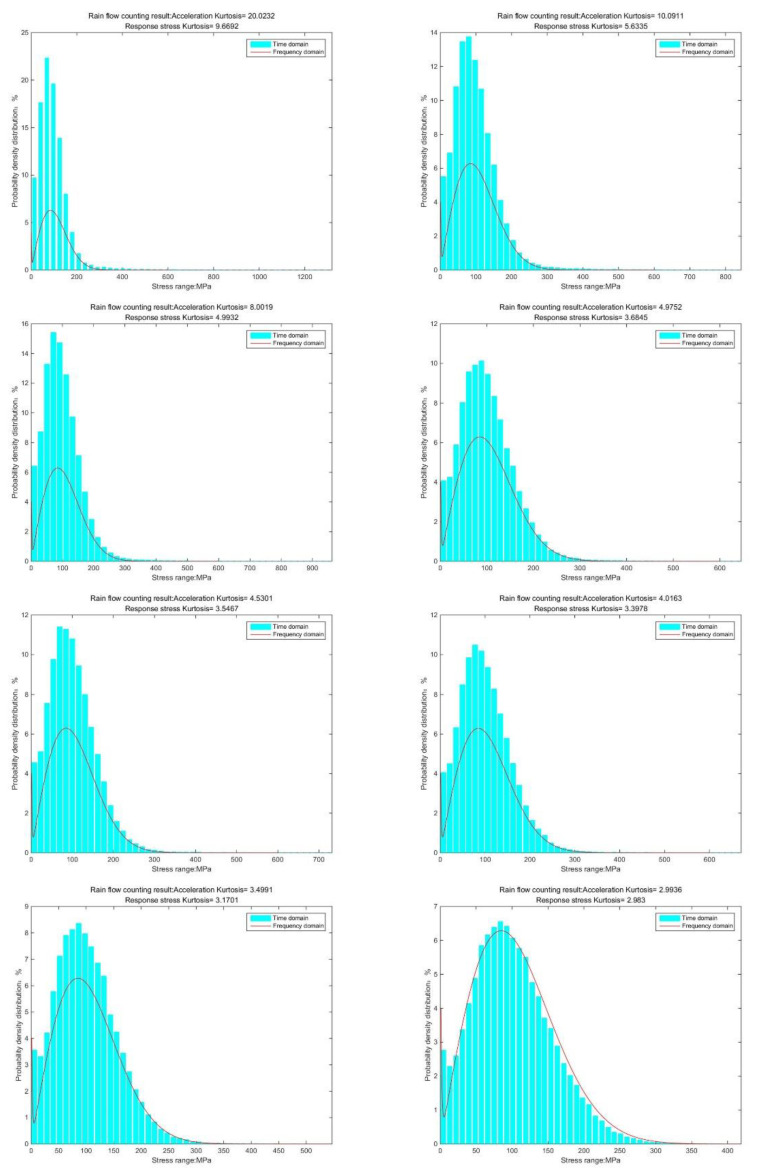
Rainflow counting results (K = 20, 10, 8, 5, 4.5, 4, 3.5, 3).

According to the results shown in Figure 6, it can be clearly observed that when the kurtosis is three, the results obtained by the time-domain method and the frequency-domain method are the same. The result from the frequency-domain method clearly finds the effect of kurtosis on the stress-cycle distribution [32].

According to Kihm’s research, the peak-to-valley value of non-Gaussian signals has a corresponding proportional relationship with RMS [15]. And it is also resented in this study, as shown in Table 5. Therefore, adjusting the rainflow counting results obtained in the frequency-domain method and obtaining the stress cycle distribution results at the corresponding kurtosis according to the kurtosis, stress RMS value and the Wohler’s curve should be considered. Next, based on the yield limit of the material, a decision is made as to whether to consider the plastic strain portion and then the damage of the elastic strain and the plastic strain are calculated separately. Finally, the expected total damage at the current kurtosis is obtained. Of course, in the actual signal, the distribution of high-stress areas is very random due to the length and low probability. Considering that the zero-mean random signal is symmetric on both sides of the 0 point, the probability and magnitude of positive and negative occurrences are equal, and thus the trend of peak and valley values with kurtosis is considered to be the same. The difference *Peak_k_* of the peak and valley values is used. Then, only a curve equation is calculated here to fit the ratio of *Peak_k_* to RMS.

According to the contour of the curve, the double exponent is selected as the basis function.

(20) $$p\frac{Peak_{k}}{RMS}=12.5*exp\left(0.0225*\left(k-3\right)\right)-7.5*exp\left(-0.75*\left(k-3\right)\right)\;$$

**Figure 7:**
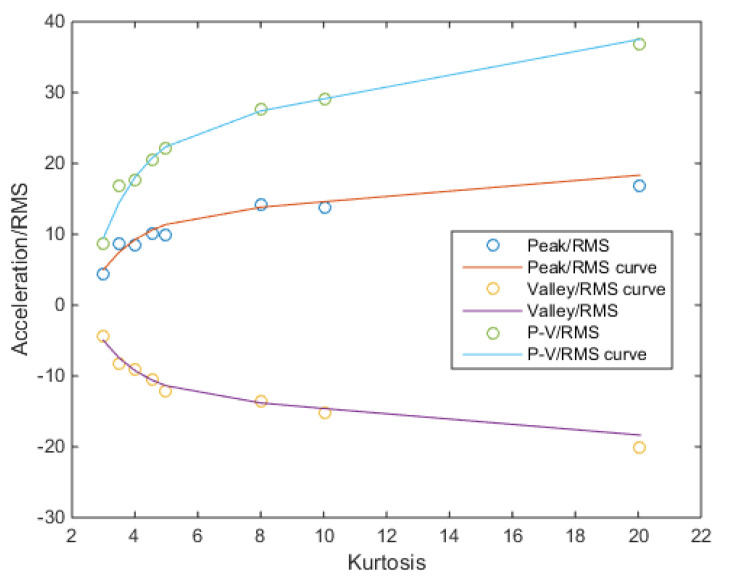
Acceleration-range fit curve by kurtosis.

Next, the stress response function of the structure could be used to obtain the stress at different acceleration values according to Figure 7. The changes in the distribution of the rain flow counting results should be considered in the next stage. Mainly focus on the center offset, the maximum distribution rate and the concentrate change.

The stress probability distribution of the structure in the frequency range and the RMS value of the stress could be calculated by the PSD approach. The max distribution position will move to the negative direction of the x-axis while kurtosis is increasing. The specific value is related to the maximum stress range. It can be known from the previous description that all signal’s mean and root mean square is the same. For the random process with different kurtosis, if the first-order or second-order statistic is constant during the growth of the maximum distribution range, as the kurtosis grows, the stress value of the maximum distribution rate will decrease, and the distribution rate of the maximum distribution will increase. So, the degree of increase in both the offset and the distribution rate based on the maximum distribution range can be inversely found. According to the characteristic of the Gaussian distribution, the change of the main distribution interval can be calculated, which refers to the distribution range of 1σ stress.

In fact, the difference in offset is not very obvious at low kurtosis. At the same time, using the ratio of the maximum probability density distribution under different kurtoses to the maximum probability distribution under the Gaussian signal, the growth rate of the rainflow counting result could be determined. All coefficient fittings are performed using the double-exponential function as the basis function.

Center-position offset fit by kurtosis:

(21) $$\Delta S_{c}=5.7*10^{4}*exp\left(-0.03010\;*\left(k-3\right)\right)-5.7*10^{4}*exp\left(-0.03012\;\;*\left(k-3\right)\right)$$

Enlarged-scale fit by kurtosis:

(22) α=1.048∗exp(0.00651∗(k−3))−0.04663∗exp(−0.6225∗(k−3))

Select the middle part of the curve and distribute the same numerical points to investigate the change of the curve span, here the value of 33% was chosen.

Enlarged scale fit by kurtosis:

(23) β=0.006886∗exp(−1.042∗(k−3))−0.9922∗exp(−0.003538∗(k−3))

Then, according to this coefficient, combined with Equation (18), the stress distribution density of any kurtosis at this node in the model under the same PSD form can be obtained.

**Figure 8:**
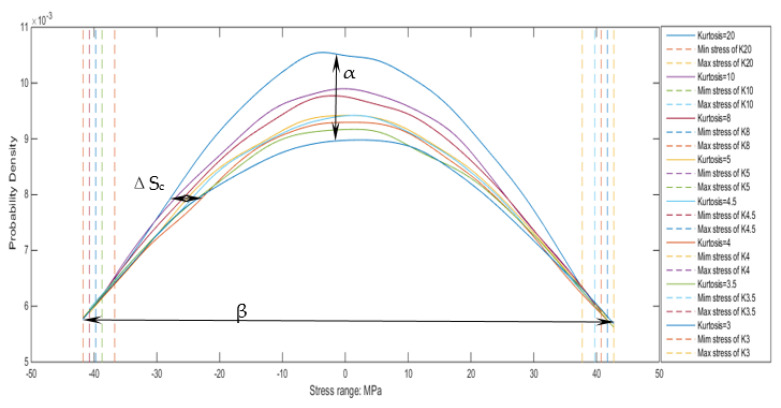
The stress distribution rate in ±RMS.

Next, fit Figure 8 to obtain the relationship between the offset of the center position and the enlarged scale with kurtosis. In this process, it could be found that between 2 RMS stress and 6 RMS stress (86.4~259.2 MPa), the distribution of non-Gaussian signals is actually smaller than the distribution of Gaussian signals. So set within this interval, use a negative offset. This reduction factor is determined by the total number of cycles. Since the total number of cycles is constant, the number of cycles obtained by enlarging the curve growth should be subtracted within this stress range. The curve is processed using a smoothing function when kurtosis is higher than 5.

Now we could infer the rainflow counting results of non-Gaussian signals based on the rainflow counting results obtained from the frequency-domain method and Gaussian signals.

To check the results, use the Bendat method in the narrow-band and the Dirlik method in the wide-band, respectively, as the fundamental equations which are modulated by using the center position offset and the enlarged scale.

So in the stress range 0–*p* RMS:

(24) $$p{\left(S\right)}^{nGNB}=\alpha *\frac{\beta \left(S-\Delta S_{c}\right)}{\sigma_{RMS}{}^{2}}exp\left(-\frac{\beta {\left(S-\Delta S_{c}\right)}^{2}}{2\sigma_{RMS}{}^{2}}\right)$$

(25) $$p{\left(S\right)}^{nGDK}=\frac{\alpha }{2\sqrt{m_{0}}}\left[\frac{G_{1}}{Q}exp\left(-\frac{Z}{Q}\right)+\frac{G_{2}Z}{R^{2}}exp\left(-\frac{Z^{2}}{2R^{2}}\right)+G_{3}Z\cdot exp\left(-\frac{Z^{2}}{2}\right)\right]$$

where *Z* is the normalized amplitude and *Xm* is the mean frequency, as fellow

$$Z=\frac{\beta \left(S-\Delta S_{c}\right)}{2\sqrt{m_{0}}}\;\mathrm{and}\;x_{m}=\frac{m_{1}}{m_{0}}{\left(\frac{m_{2}}{m_{4}}\right)}^{\frac{1}{2}}$$

Other parameters are defined as:

$$G_{1}=\frac{2\left(x_{m}-\alpha_{2}^{2}\right)}{1+\alpha_{2}^{2}},$$

$$G_{2}=\frac{1-\alpha_{2}-G_{1}+G_{1}^{2}}{1+R},$$

$$G_{3}=1-G_{1}-G_{2},$$

$$R=\frac{\alpha_{2}-x_{m}-G_{1}^{2}}{1-\alpha_{2}-G_{1}+G_{1}^{2}}\;\mathrm{and}\;Q=\frac{1.25\left(\alpha_{2}-G_{3}-G_{2}R\right)}{G_{1}}$$

**Figure 9:**
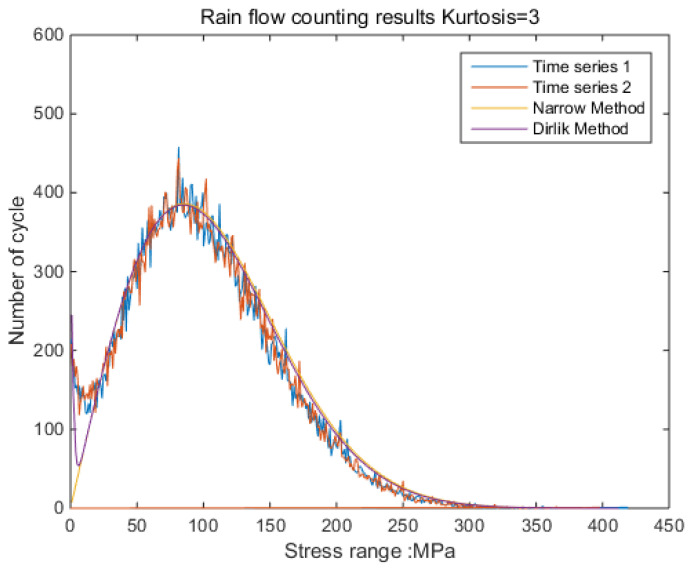
Rainflow counting results for Kurtosis = 3.

**Figure 10:**
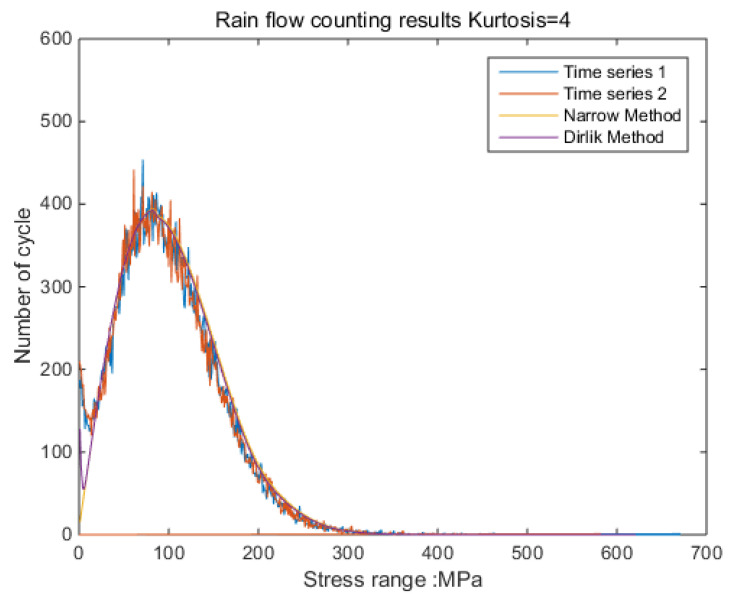
Rainflow counting results for Kurtosis = 4.

**Figure 11:**
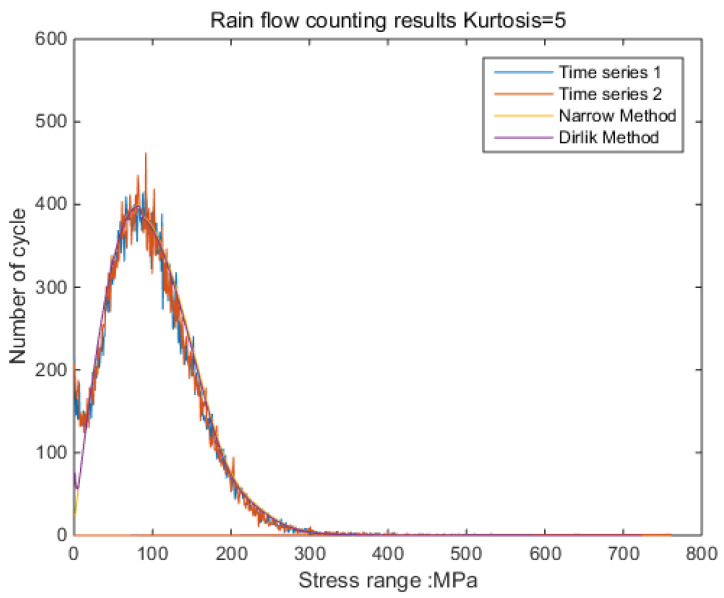
Rainflow counting results for Kurtosis = 5.

**Figure 12:**
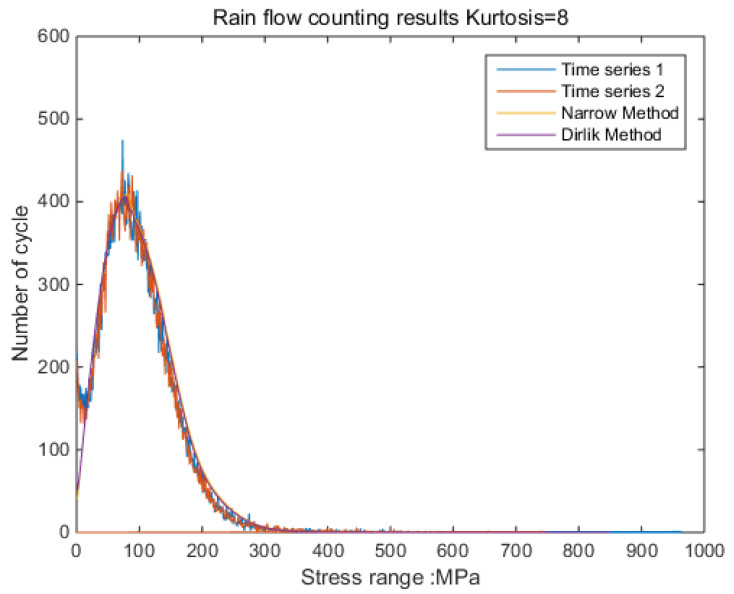
Rainflow counting results for Kurtosis = 8.

**Figure 13:**
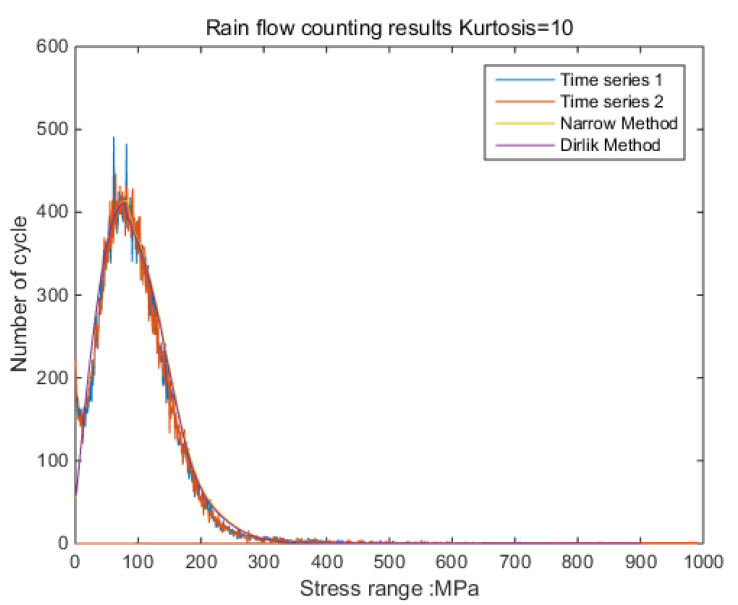
Rainflow counting results for Kurtosis = 10.

**Figure 14:**
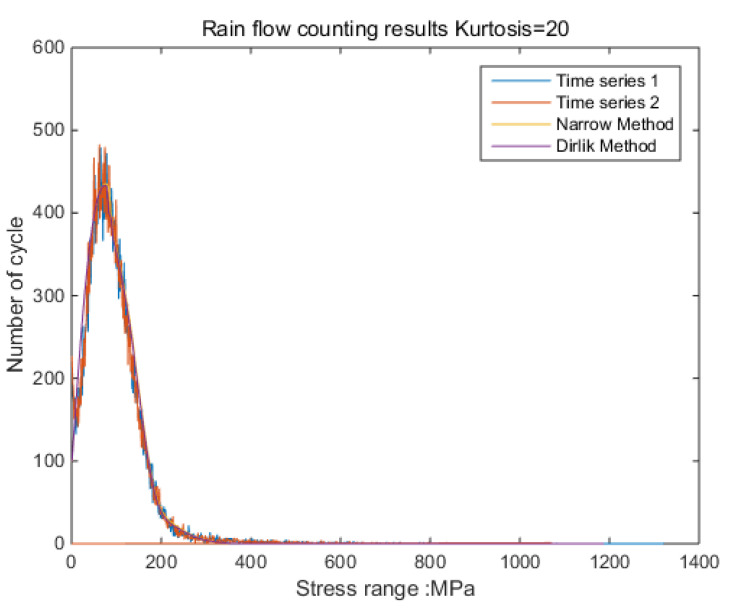
Rainflow counting results for Kurtosis = 20.

The new results by kurtosis increase comparison with time series as follows: by the moving-average method, the new results look similar to the original rainflow counting results. The results for a total of 5 kurtosis levels are presented in Figure 9, Figure 10, Figure 11, Figure 12, Figure 13 and Figure 14.

The actual operation method is to sum according to the probability density distribution function, which is usually around 98%, and the remaining 1–2% is evenly supplemented on 6RMS–pRMS. Next, it is only necessary to calculate according to the damage-estimation method proposed previously. Errors are unavoidable, especially for random signals; only approximate damage can be estimated. Each adjustment coefficient and the degree of attenuation of the kurtosis are affected by the material properties and the structure of the specimen. Therefore, before applying, the coefficients of the specific structure need to be verified by actual experiments.

**Figure 15:**
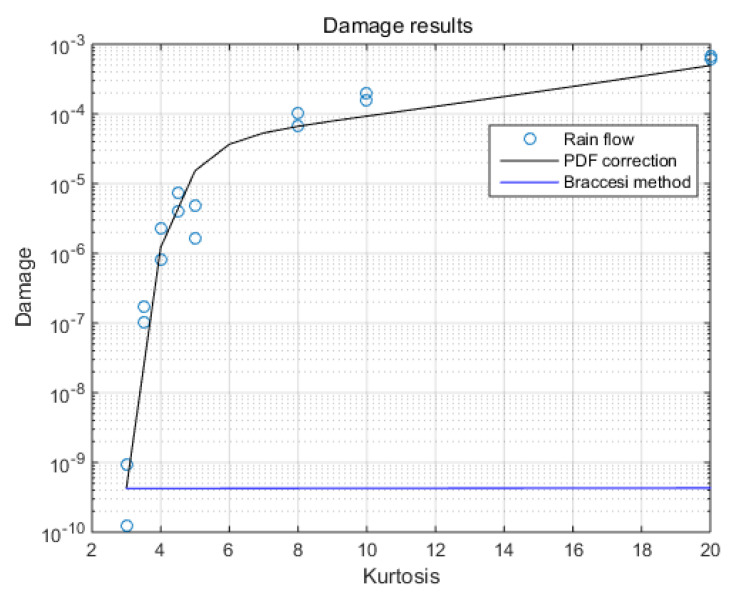
Damage results in 10 min.

By comparing the damage with the different kurtoses levels from the time domain method, the results obtained by Braccesi’s method and the new method are compared, shown in Figure 15. The considerable difference may come from Wohler’s curve of the material.

The advantage of this method is that there is no need to simulate the stress time series, but the distribution of the excitation time series needs to be made. However, the probability density distribution function has a smaller probability value in a high-stress range. When the size of data cannot meet the requirements, the results are greatly affected by randomness.

**Figure 16:**
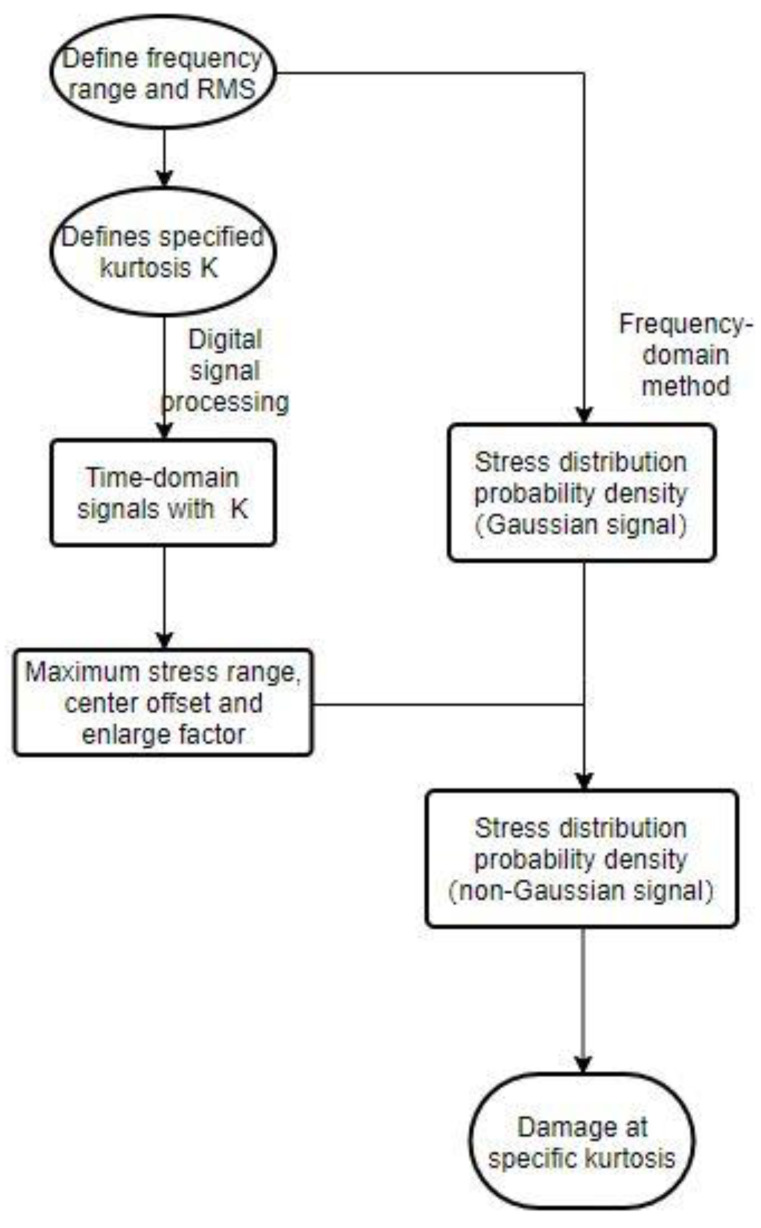
Corrective method flowchart.

So, this method can be summarized as follows: first, according to the requirements, establish the time-domain signal under the demand kurtosis. Through the frequency-domain method, the probability distribution of the structural response stress of the material in a specific frequency range under the Gaussian signal is obtained. The corresponding relationship between acceleration and stress at the point of interest is obtained using experimental or simulation methods. The fitting method was used to infer the maximum value of the stress range, the maximum distribution position and the magnification factor. Finally, the stress probability density function under the Gaussian signal obtained by the frequency domain method is used to infer the stress-probability density function under the non-Gaussian signal at a specific kurtosis for damage estimation. It should be pointed out that the form of the coefficient is not unique; only the one with better adaptability is selected this time. The flowchart is as follows in Figure 16.

## 5. Verification Experiment

**Figure 17:**
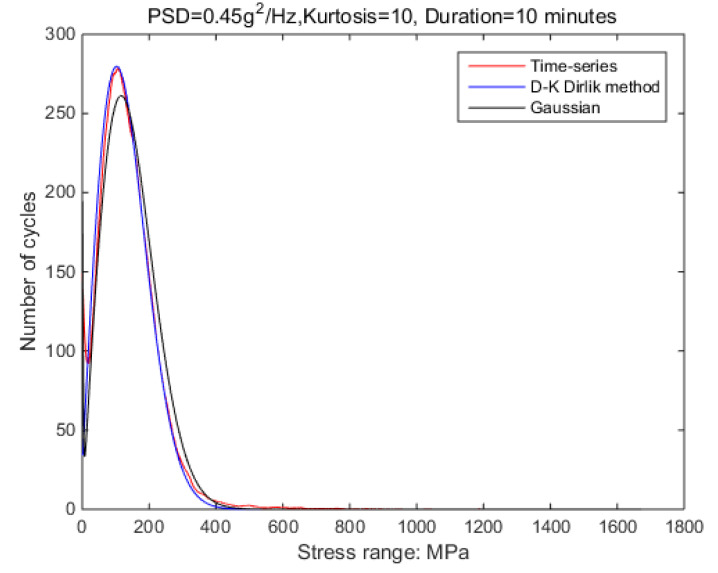
Comparison of time-domain and frequency-domain results.

**Figure 18:**
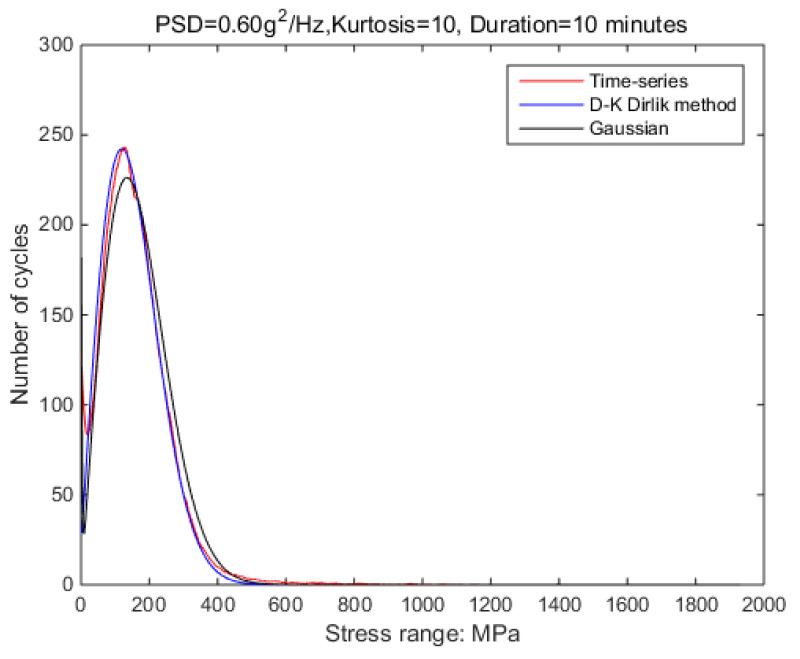
Comparison of time-domain and frequency-domain results.

In order to verify the validity of this result, the following signals are used for further verification; details are shown in Table 6. In this process, the length and frequency range of the non-Gaussian signal and PSD shape are consistent with those previously used as illustrated in the Figure 17 and Figure 18. In this case, 0.4 g^2^/Hz is the highest load that the shaker can accept when kurtosis is 10. As the PSD is increasing, the shaker would overload. However, in the case of Gaussian random vibration, this situation will not occur.

A totally new specimen is applied to non-Gaussian random vibration excitation until failure by the shaker. In the process, the failure standard of the virtual fatigue test is based on the failure of the node; thus, ΣD = 1. The actual fatigue test standard is based on the appearance of visible cracks at critical locations. According to the established finite element virtual model, the load of the same level is applied to Abaqus, and the response stress of the critical node is recorded.

**Figure 19:**
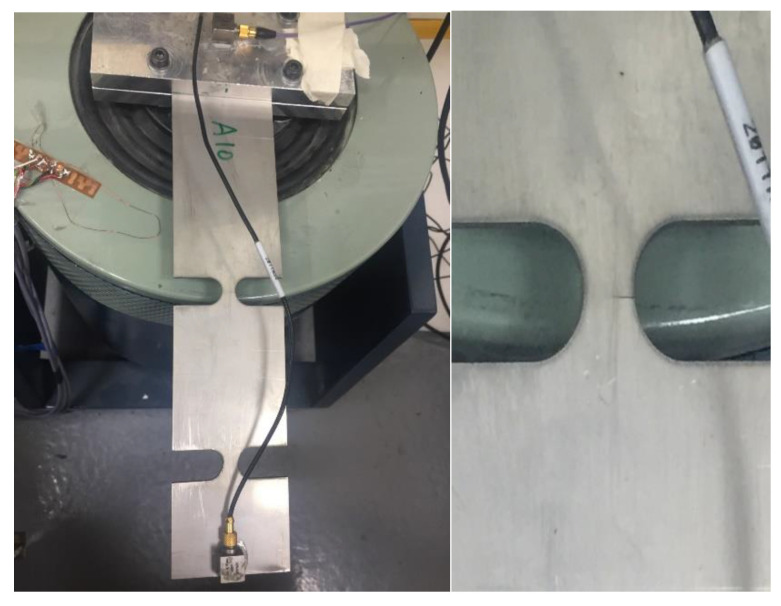
Non-Gaussian fatigue test.

After 1.5 h, visible cracks appeared in the middle of the first notch of the specimen. The location of the crack generation is shown in Figure 19, which is consistent with the expected location. In fact, the lifetime of Gaussian random vibration at a PSD of 0.7 g^2^/Hz is the same as the above damage. It is clear that the harm of kurtosis is more worthy of attention.

**Figure 20:**
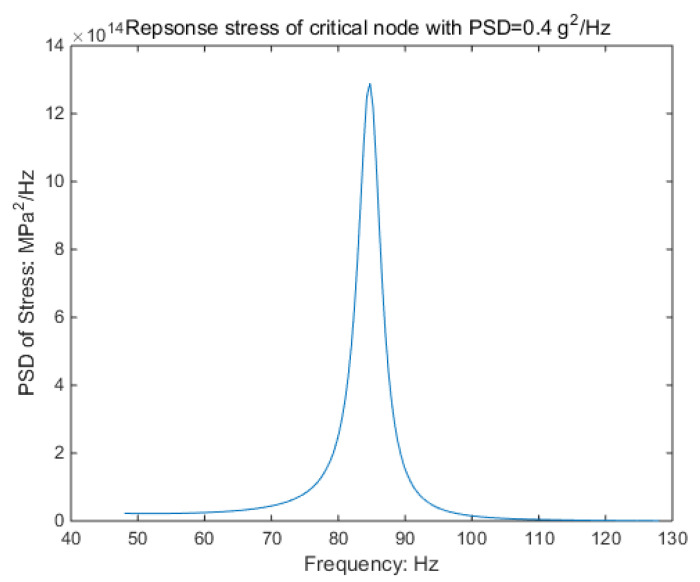
Response stress of the PSD approach.

**Figure 21:**
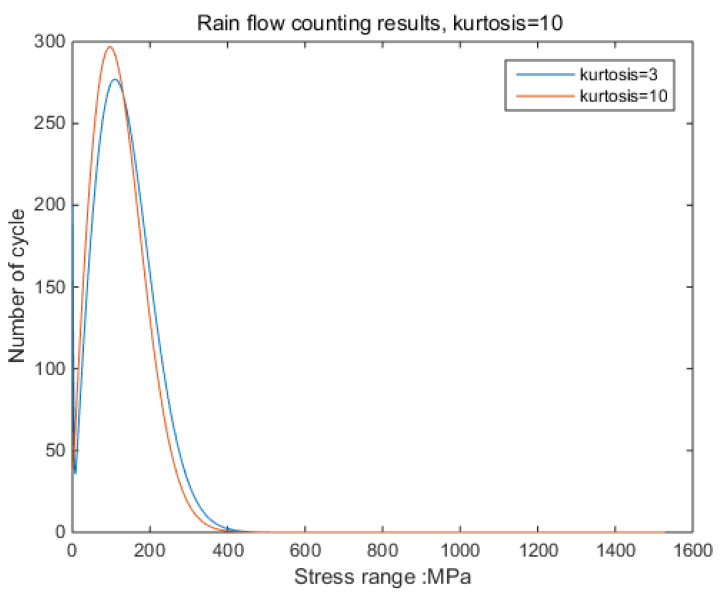
Rainflow result of kurtosis = 10.

In order to reduce the simulation time, the duration time of the input signal is selected as 10 min. The non-Gaussian random vibration excitation with the same characteristics as the experiment is applied to the virtual specimen. By recording the response stress of critical nodes within 10 min, the damage value during this period can be estimated; the process is shown in Figure 20 and Figure 21.

According to the material property and the above rainflow counting results, it can be calculated that the damage is about 0.067 within 10 min. Therefore, when the damage is equal to 1, the life of the specimen is 149.25 min or 2.48 h. With the same PSD level, the average life of the Gaussian signal is 71.4 h. The results of other methods are shown in Table 7. Relatively speaking, this method further improves the accuracy of the calculation.

## 6. Discussion of Results

By comparing the rain count results, three fundamental parameters should be focused on: maximum stress, maximum distribution value and location of the maximum distribution.

By Figure 9, Figure 10, Figure 11, Figure 12, Figure 13 and Figure 14, it is confirmed that the new correction method can well infer the fatigue damage of the structure under different kurtosis signal excitations with the same PSD level. A comparison using two methods as basic functions, Dirlik and Bendat, reveals little difference. This means that this correction can be applied to both narrow-band and wide-band. Unfortunately, this study was not available to validate wide-band.

In this method, the strain-stress transfer function of the structure is used. With Figure 17 and Figure 18, it can be confirmed that this transfer function is only related to the structure. When the structure is constant, the results obtained by this method can be applied to different PSD classes. That is, it is possible to use low PSD levels to obtain a correction relationship to solve fatigue damage for high PSD levels. Note that random vibrations with high PSD peaks have very high equipment requirements for laboratory simulations. However, using this method, the study will become possible.

Based on the validation tests, it can be determined that the method further improves the accuracy of damage calculation, shown in Table 7. At the same time, it can be found that a larger kurtosis has a significant effect on fatigue damage, making the fatigue damage under excitation at low PSD levels reach the damage under Gaussian excitation at high PSD levels.

## 7. Conclusions

In this paper, the dynamic behavior of double-notch specimens was studied in order to find how the kurtosis of the excitation affects the response stress. The relation of kurtosis with the excitation and response stress of the critical position is found, which makes fatigue estimation with excitation kurtosis possible.

In fact, the new method has good performance under high kurtosis, which was proved by experiments. Additionally, it applies to all PSD levels and is not limited to the PSD level used to obtain the model. So, it is possible to predict fatigue damage at a high PSD level by using a lower PSD level in the same structure, especially when the test conditions are limited. At present, this method can be close to the real situation in high kurtosis; the accuracy now is 66%, much better than the previous. Both the fitting method and the choice of basis function affect accuracy. In this paper, the more usable double exponential function model is used. However, this result can be further optimized.

## Figures and Tables

**Table 1:** Material card of AISI304.

Density	Elastic Modulus	Yields Strength	Ultimate Tension Strength	m
7942.2 kg/m^3^	161 GPa	365 MPa	710.5 MPa	17.92

**Table 2:** Frequency-domain information (g = 9.81 m/s^2^).

Frequency (Hz)	PSD (g^2^/Hz)	PSD (m^2^/s^4^)/Hz
48	0.25	24.05
128	0.25	24.05
RMS	4.472136 g	43.87 m/s^2^

**Table 3:** RMS and Kurtosis of excitation signals.

Duration	10 min		Sample Rate		512			
No.	1	2	3	4	5	6	7	8
RMS (m/s^2^)	43.84	43.73	43.98	43.76	43.81	43.92	43.83	43.83
Kurtosis	20.02	20.08	10.09	10.04	8.00	8.00	4.98	4.98
No.	9	10	11	12	13	14	15	16
RMS (m/s^2^)	43.71	43.82	43.93	43.84	43.88	43.84	43.85	43.85
Kurtosis	4.53	4.55	4.02	4.02	3.50	3.50	2.99	3.00

**Table 4:** The RMS and Kurtosis of response stress.

Duration	10 min		Sample Rate		512			
No.	1	2	3	4	5	6	7	8
RMS (MPa)	43.32	43.02	43.31	43.02	43.07	43.05	43.17	43.08
Kurtosis	9.67	9.49	5.63	5.69	4.99	4.84	3.68	3.69
No.	9	10	11	12	13	14	15	16
RMS (MPa)	42.97	43.13	43.19	43.15	43.12	43.12	43.10	43.14
Kurtosis	3.55	3.56	3.40	3.38	3.17	3.19	2.98	2.98

**Table 5:** Accelerated statistical comparison among different kurtoses.

Kurtosis	20.06	10.06	8.00	4.98	4.54	4.02	3.50	3.00
Mean (m/s^2^)	0	0	0	0	0	0	0	0
RMS (m/s^2^)	43.78	43.87	43.86	43.83	43.77	43.88	43.86	43.85
Peak/RMS	16.88	13.87	14.13	9.96	10.05	8.57	8.63	4.44
Valley/RMS	−20.05	−15.16	−13.52	−12.20	−10.47	−9.07	−8.25	−4.30
P-V/RMS	36.93	29.03	27.65	22.16	20.51	17.64	16.88	8.73

**Table 6:** Input loading in the verification experiment.

PSD Level	Kurtosis	Corrective Method (Numerical)	Virtual Experiment (Time Domain)	Experiment
0.45 g^2^/Hz	10	10 min	10 min	
0.6 g^2^/Hz	10	10 min	10 min	
0.4 g^2^/Hz	10	10 min	10 min	Failure

**Table 7:** The result of the fatigue of validation experiment.

PSD Level	Kurtosis	Corrective Method	Braccesi’s	Nieslony’s	Experiment
0.45 g^2^/Hz	10	2.48 h	70.9 h	66.9 h	1.5 h

## Data Availability

Not applicable.
